# Supplementary material for: The cost of illness for childhood clinical pneumonia and invasive pneumococcal disease in Nigeria
Source: BMJ Glob Health. 2022 Jan 31;7(1):e007080. doi: 10.1136/bmjgh-2021-007080 (PMC8804652; doi:10.1136/bmjgh-2021-007080)
Supplement: Supplementary data [file bmjgh-2021-007080supp001.pdf]

SUPPLEMENTARY MATERIAL

Table S1: Unit cost estimates and sources for parameters used in cost analysis in (US$ 2020)

Service	Unit cost US$		Source
	AKTH	MMSH	
Chest X-ray	6.9	4.2	Radiology department
CSF chemistry, microscopy and culture	2.2	1.7	Microbiology department
Blood culture	13.9	3.3	Microbiology department
Full blood count	1.5	1.4	Haematology department
Haemoglobin	0.8	0.6	Haematology department
Urea and electrolyte	3.6	1.9	Chemical pathology department
Malaria parasite	1.1	0.6	Haematology department
Blood transfusion	13.9	13.9	Haematology department
Oxygen	20.0	10.0	Paediatric ward
Bed day AKTH (full cost)	55.5	33.3	Paediatric ward AKTH
Bed day (WHO-CHOICE)	29.8	16.7	WHO-CHOICE
OPD visit (WHO-CHOICE)	7.8	7.7	WHO-CHOICE

Table S2: Description of children and caregivers[1]

	Outpatient pneumonia N=244	Inpatient pneumonia N=117	Septicaemia N=66	Meningitis N=53
**Hospital n (%)**				
AKTH	135(57.2)	48(41.0)	32(48.5)	21(39.6)
MMSH	109(44.9)	69(59.0)	34(51.5)	32(60.4)
**Child characteristics**				
**Age in months**				
Median (IQR)	17.9 (12-27)	16.3 (12-26)	24.2 (16-36)	24.6 (18-31)
**Age group(months) n (%)[1]**				
<1-11	57 (23.4)	25 (21.4)	6 (9.1)	5 (9.4)
12-23	98 (39.7)	58 (49.6)	26 (39.4)	19 (35.9)
24+	90 (36.9)	34 (29.1)	34 (51.5)	29 (54.7)
**Gender, Female n (%)**	119 (48.8)	53 (45.3)	31 (47.0)	23 (43.4)
**Prior care sought n (%)**				
None	72 (29.5)	25 (21.4)	15 (22.7)	6 (11.3)
Private hospital	38 (15.6)	17 (14.5)	19 (28.8)	22 (41.5)
Chemist	106 (43.4)	60 (51.3)	28 (42.4)	18 (34.0)
Others	28 (11.5)	15 (12.8)	4 (6.1)	7 (13.2)
Missing				
**Caregiver characteristics[1]**				
Age in years, median (IQR)	28.0 (27-30)	29 .0 (28-30)	29.0 (28-30)	30.0 (28-31)
Relationship to child, Mother	224 (91.8)	115 (98.3)	66 (100.0)	49 (92.5)
**Highest education of caregiver[1] n (%)**				
None	20 (8.2)	7 (6.0)	0 (0.0)	1 (1.9)
Primary	19 (7.8)	7 (6.0)	2 (3.0)	3 (5.6)
Secondary	88 (36.1)	48 (41.0)	34 (51.5)	18 (34.0)
Tertiary	117 (47.9)	52 (44.4)	29 (43.9)	31 (58.5)
Missing	0 (0.0)	3 (2.6)	1 (1.5)	0 (0.0)
**Occupation of caregiver[1] n (%)**				
Self-employed	98 (40.2)	54 (46.1)	30 (45.5)	20 (37.7)
Salaried work	97 (39.7)	56 (47.9)	36 (54.5)	32 (60.4)
Unemployed	49 (20.1)	7 (6.0)	0 (0.0)	1 (1.9)

[1] Main caregiver

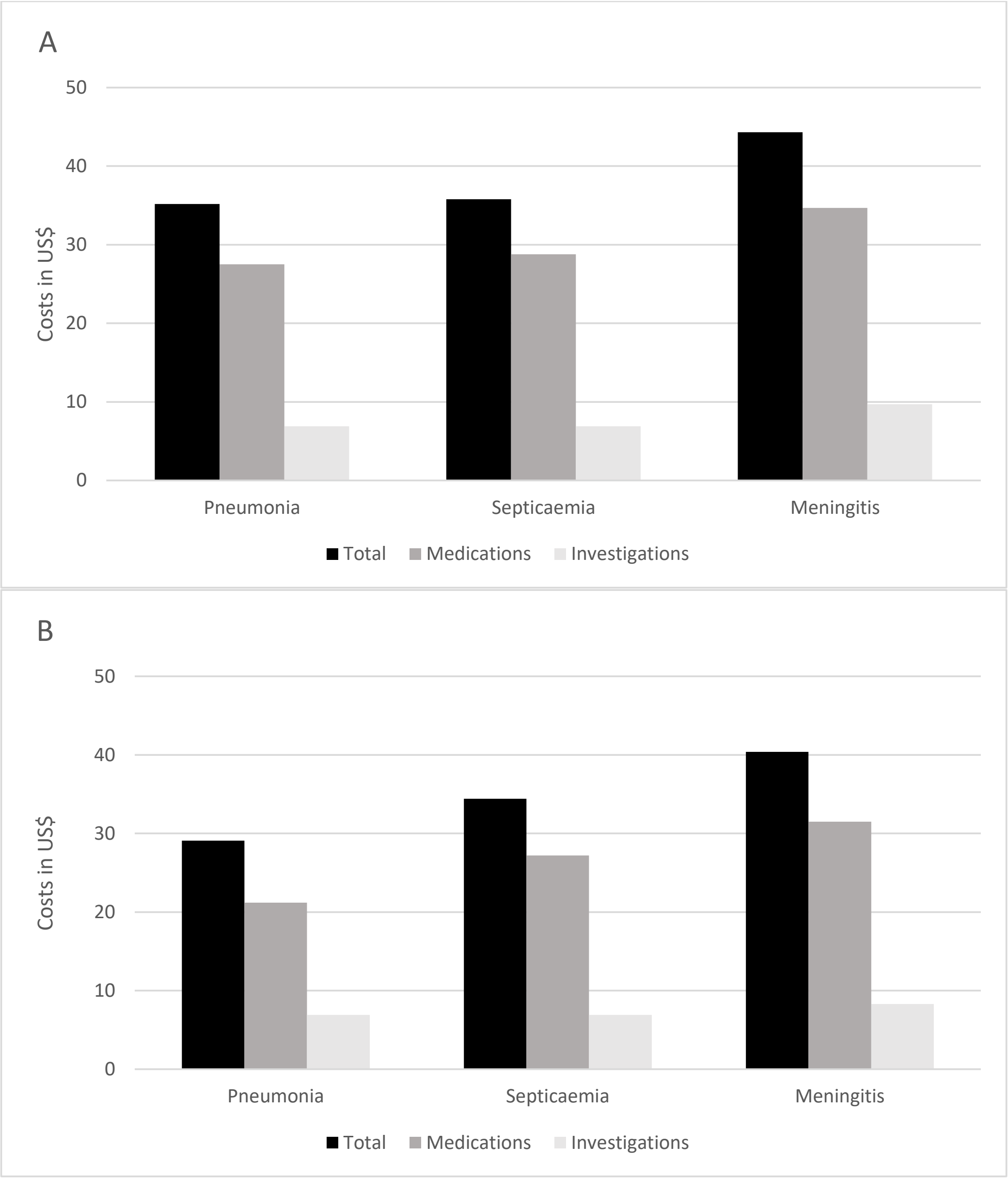

Figure S1: Breakdown of User fees for AKTH (A) and MMSH (B)

Table S3 : Costs incurred by households stratified by income tertiles

	Household costs as % of monthly household income					
	Tertile 1(poorest)		Tertile 2		Tertile 3 (least poor)	
	Mean (SD)	Median (IQR)	Mean (SD)	Median (IQR)	Mean (SD)	Median (IQR)
**Pneumonia**						
Direct cost	34 (62)	21 (17-29)	18 (5)	17 (14-20)	13 (4)	13 (10-15)
Indirect cost	7 (5)	7 (5-9)	7 (3)	7 (5-8)	6 (3)	6 (4-8)
Total household cost	41 (61)	29 (23-39)	25 (5)	25 (22-28)	19 (5)	19 (15-22)
**Septicaemia**						
Direct cost	40 (66)	27 (20-35)	37 (56)	21 (16-33)	16 (5.40)	16 (12-20)
Indirect cost	9 (4)	8 (6-12)	9 (3)	8 (7-10)	7 (4)	6 (4-9)
Total household cost	49 (68)	35 (28-42)	46 (58)	29 (24-44)	24 (8)	21 (17-29)
**Meningitis**						
Direct cost	66 (120)	33 (30-42)	25 (8)	25 (21-30)	16 (5)	16 (13-20)
Indirect cost	14 (21)	9 (6-11)	10 (4)	9 (8-10)	9 (4)	8 (6-10)
Total household cost	79 (140)	42 (37-53)	34 (9)	34 (25-41)	24 (6)	24 (22-29)

Table S4: Senistivity analyses for provider costs in US$ using WHO-CHOICE estimates of bed-day costs and WTP approach for indirect costs

	Costs US$						
	Inpatient pneumonia		Septicaemia		Meningitis		P value
	Mean (SD)	Median (IQR)	Mean (SD)	Median (IQR)	Mean (SD)	Median (IQR)	
**AKTH**							
Provider costs (WHO-CHOICE)							
Bed day	146 (24)	149 (119-149)	159 (38)	149 (148-179)	189 (32)	179 (178-208)	<0.001
Total provider costs	223 (44)	219 (189-244)	217 (74)	197 (172-238)	257 (56)	253 (226-295)	0.007
Indirect costs							
WTP	25 (22)	24 (14-31)	41 (51)	28 (14-53)	56 (36)	39 (33-83)	<0.001
**MMSH**							
Provider costs (WHO-CHOICE)							
Bed day	95 (55)	84 (83-100)	93 (25)	84 (83-117)	119 (81)	100 (84-117)	0.004
Total provider costs	148 (63)	137 (120-153)	135 (44)	124 (108-159)	179 (98)	156 (131-174)	0.001
Indirect costs							
WTP	52 (104)	21 (13-50)	27 (25)	20 (14)	73 (134)	33 (28-74)	0.009
